# Age- and sex-dependent effects of stressors on activity of the nucleus reuniens of the thalamus

**DOI:** 10.1016/j.schres.2025.09.018

**Published:** 2025-09-26

**Authors:** Daniela L. Uliana, Anthony A. Grace

**Affiliations:** a Departments of Neuroscience, Psychiatry and Psychology, University of Pittsburgh, Pittsburgh, PA, USA

**Keywords:** Nucleus reuniens of thalamus, Sex, Stress, Puberty

## Abstract

**Background:**

Stress is a significant socio-environmental risk factor for schizophrenia, with its impact varying with age and sex. Male rats are more vulnerable to the long-term effecct of stress during early adolescence, whereas females are more affected during late adolescence, with both demonstrating a stress-induced hyperdopaminergic state and ventral hippocampal hyperexcitability. The nucleus reuniens of the thalamus (RE) plays a crucial role in modulating hippocampal-prefrontal connectivity and dopamine activity. This study investigated the effect of stress during neurodevelopment on RE activity in both sexes.

**Study design:**

Sprague-Dawley rats were subjected to a 10-day footshock and restraint stress protocol during early adolescence (Post-natal day [PD] 31–40) or late adolescence (PD41–50) periods. Electrophysiological RE recordings were conducted 1–2 and 5–6 weeks post-stress.

**Study results:**

Early adolescence stress did not affect the number of spontaneously active RE neurons in males and females after 1–2 or 5–6 weeks, but it increased the proportion of RE neurons firing in bursts in females. Late adolescence stress increased the number of spontaneously active RE neurons in females at both 1–2 and 5–6 weeks. Females had fewer active RE neurons than males starting earlier in adulthood but not at a younger age (PD47–54). This shows an age-dependent effect on female RE activity.

**Conclusion:**

Stress had sex-specific effects on RE neuron activity of females, with late adolescence stress increasing the number of spontaneous RE neurons, while early adolescence stress influenced burst firing. Therefore, stress-induced changes in RE activity during adolescence may contribute to females’ vulnerability to neuropathology.

## 1. Introduction

Stress is a socio-environmental risk factor for different psychiatric disorders, such as major depressive disorder and schizophrenia (Kendler et al., 1999; Pruessner et al., 2011). One factor that could be a determinant to the outcome is age of exposure (Solmi et al., 2022; Thapar and Riglin, 2020). For example, childhood trauma occurring during the adolescence shows a stronger association with an increased risk of psychosis (Croft et al., 2021, 2019). In contrast, trauma experienced during adulthood is more strongly linked to the development of affective disorders (Marshall, 2016). Early life adversity significantly impacts the development of neuropathological states, with males and females having different vulnerability periods (Hodes and Epperson, 2019). Extensive evidence demonstrates sex differences in stress responses, from initial neuroendocrine activation to the reorganization of stress-sensitive neural circuits (Wellman et al., 2018). In fact, rodent stress models are designed to induce a similar overall degree of physiological activation as observed in humans, which includes HPA axis activation, amygdala activation, and hypervigilance (Atrooz et al., 2021; Daviu et al., 2024; Lovelock and Deak, 2019). For example, footshock and restraint stress are commonly used in the preclinical field to elicit these physiological responses and model the adversity experienced by humans. Using a combination of footshock and restrain stress, we have described that male rats are sensitive to stress during early stress (Postnatal day, PD31–40) and females to late adolescence stress (PD41–50) in inducing adult hyperdopaminergic states in the ventral tegmental area (VTA) (Zhu and Grace, 2022). In fact, this increased dopaminergic activity is a core neurobiological dysfunction in schizophrenia proposed to underlie the positive symptoms (Kesby et al., 2018).

Male and female susceptibility to a hyperdopaminergic state in their respective periods of susceptibility is driven by a higher excitability state of the ventral hippocampus (vHipp) likely due to parvalbumin loss (Zhu and Grace, 2022). Considering the connectivity of vHipp with the amygdala, male susceptibility also was linked to increased basolateral amygdala activity, an event that was not observed in females subjected to late adolescence stress (Zhu and Grace, 2022). Therefore, the precise neurobiological mechanisms that may contribute to vHipp hyperexcitability and females’ susceptibility are still unclear.

One thalamic area that has connectivity with vHipp and is shown to modulate DA activity through the vHipp is the nucleus reuniens of the thalamus (RE) (Zimmerman and Grace, 2016). Indeed, RE dysregulation has been proposed to contribute to the circuit dysfunction observed in schizophrenia (Dolleman-van der Weel and Witter, 2020; Duan et al., 2015), considering that is an important area regulating cognitive and executive function (Dolleman-van der Weel et al., 2019; Rahman et al., 2021; Viena et al., 2018). RE integrates frontocortical areas with the hippocampus (Di Prisco and Vertes, 2006; Herkenham, 1978; Vertes, 2002; Zhang et al., 2012; Zimmerman and Grace, 2016) which enables prefrontal cortex (PFC) modulation of the vHipp (Griffin, 2015; Varela et al., 2014; Vertes et al., 2007). Hippocampal-prefrontal connectivity disruption is also described in schizophrenia in which RE plays a critical relay role (Dolleman-van der Weel and Witter, 2020; Godsil et al., 2013; Small et al., 2011). However, it remains unclear whether sex influences intrinsic RE activity and contributes to the observed differences in stress susceptibility between males and females. Therefore, the study aimed to evaluate the impact of stress exposure during early and late adolescence on RE activity in male and female rats.

## 2. Material and methods

### 2.1. Animals

**Fig. 1.**
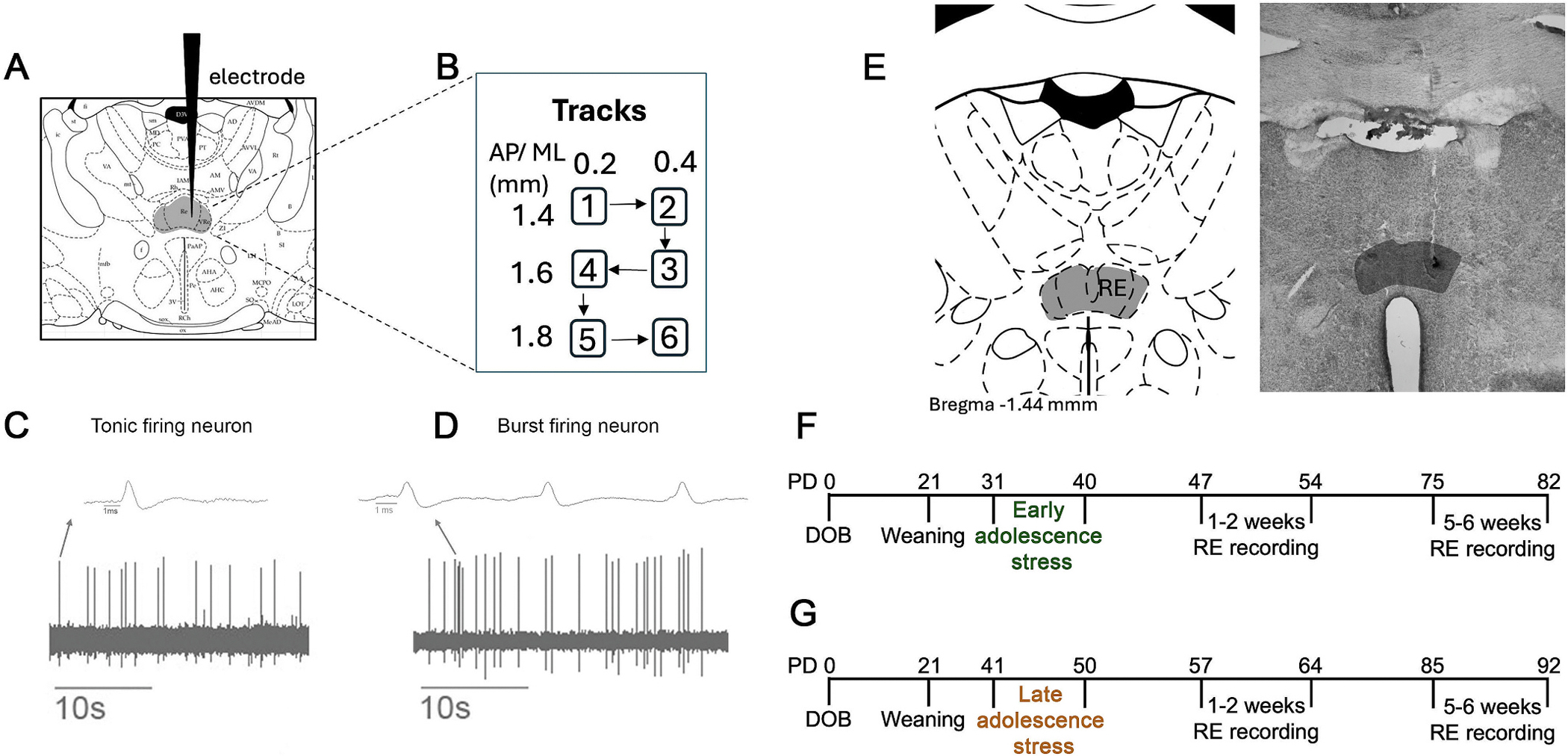
Experimental design illustrating the protocol to study the effect of early and late adolescence stress on RE activity. In vivo single-unit extracellular recording of the RE (A) was performed in a grid pattern (B). Tonic firing (C) and burst firing of RE neurons (D). A histological representation of the RE electrode trace is shown (E). Rats were born in the facility, weaned at postnatal day (PD) 21, and subjected to stress during PD31–40 for early adolescence (F) or PD41–50 for late adolescence (G). RE recordings were conducted 1–2 weeks or 5–6 weeks post-stress.

Sprague-Dawley rats of different ages were generated from pregnant females obtained at gestational day 15 from Envigo (Indianapolis, IN). All rats were housed in a temperature- (22 ± 1 °C) and humidity-controlled room, with a 12 h/light-dark cycle (7 am/7 pm) and water/food available ad libidum. Male and female offspring were weaned at PD 21 and double-housed with their littermates. Only 2 rats per dam were used per experimental group. The stress protocol and electrophysiology were performed during the lights-on cycle (early morning to mid-afternoon period). Male and female rats were submitted to the stress protocol for 10 days during early (PD31–40) or late (PD41–50) adolescence period. RE recording took place between 1 and 2 weeks and 5–6 weeks post-stress (Fig. 1F and G). All procedures were carried out in accordance with the NIH Guide for the Care and Use of Laboratory Animals and approved by the Institutional Animal Care and Use Committee of the University of Pittsburgh.

### 2.2. Stress paradigm

The stress protocol and time periods were used to match and extend our previous studies (Gomes et al., 2019a; Gomes and Grace, 2017; Klinger et al., 2019; Uliana et al., 2021). The animals underwent a daily footshock (FS) stress session (25 shocks, 1 mA for 2 s, with random intervals of 20–60 s) over a period of 10 days. FS was paired with three 1-hour restraint stress (RS) sessions, conducted in a Plexiglas cylindrical tube on days 1, 2, and 10, immediately following the FS session.

### 2.3. Electrophysiology

Electrophysiological recordings were conducted between 1 and 2 weeks or 5–6 weeks after each stress protocol (Fig. 1F and G). The recording timepoints were chosen to evaluate the short-term (1–2 weeks) and long-term (5–6 weeks) effect of early and late adolescence stress. Rats were anesthetized with an intraperitoneal injection of chloral hydrate (400 mg/kg, i.p.), and additional doses were administered as needed to maintain anesthesia (absence of the hindlimb compression reflex). The rats were secured in a stereotaxic frame (Kopf) with body temperature maintained at 37 °C using a thermostatically-controlled heating pad (TR-200, F.S.T.). In vivo extracellular recordings were performed using microelectrodes prepared from Omegadot 2.0 mm glass tubing pulled with a vertical electrode puller (Narishige P-2) and the tip broken under a microscope. The microelectrode was filled with 2 % Chicago Sky Blue dye dissolved in 2 M NaCl with an impedance of 6–16 MΩ. Signals were amplified (x1000) using a preamplifier (2400A, Dagan) and filtered with open filter settings (low-frequency cutoff: 10 Hz; high-frequency cutoff: 16 kHz). The signal was displayed on an oscilloscope (PM3337, Philips) and digitalized via a PowerLab 8/30 interface (ADInstruments) connected to a computer running LabChart v.8 software. For analysis, signals with a signal-to-noise ratio greater than 3 were used. Electrodes were lowered into the RE in 6 vertical tracks at 0.2 mm intervals in the x-y plane (Fig. 1B) via hydraulic micropositioner (Model 2650, David Kopf instruments, California). The sampling area encompassing the RE was defined relative to the bregma and dural surface (in mm) with coordinates depending on the age (PD47–64, AP −1.25 to −1.65, ML 0.2–0.4, DV −5.0–7.0; PD >65: AP −1.4 to −1.8, ML 0.1 to 0.3, and DV −5.5 to −7.5). The measures included in the analysis were the average number of principal neurons per track, firing rate (Hz), and the % of spikes in burst, as previous reported for RE single-unit excelular recordings (Zimmerman and Grace, 2018). Putative glutamatergic projection neurons were identified based on the long duration spike criteria of a trough-to-peak width of >0.4–0.5 ms, a spike duration of at least 2 ms, and an average firing rate of 2 Hz (Zimmerman and Grace, 2018). This distinguishes them from interneurons, which have a higher firing rate (typically >10 Hz), narrower spike half-widths (less than 0.3 ms), and a lower burst propensity (Likhtik et al., 2006; Rosenkranz and Grace, 1999; Zhang and Rosenkranz, 2016). The burst activity for RE neurons were identified based on the following criteria: a maximum inter-spike interval of ≤6 ms to initiate a bursts, a maximum interval of 10 ms to terminate a bursts, a minimum interval of 200 ms between bursts, a minimum burst duration of 2 ms, and at least 2 spikes per burst (Tonic firing, Fig. 1C versus Burst firing, Fig. 1D) (Kim et al., 2011; Zimmerman and Grace, 2018). All neurons included in the analysis were verified to be within the RE by comparing their location to the marked recording site (Fig. 1E).

### 2.4. Histology

Following electrophysiological recordings, electrode localization was confirmed by ejecting Chicago Blue dye using a constant negative current of −20 μA for 20 min. The brains were removed and fixed in 8 % paraformaldehyde for 48 h, then transferred to a 25 % sucrose solution for cryoprotection. Once fully saturated, the brains were frozen and coronally sectioned at 60 μm using a cryostat (Leica Frigocut 2800). Sections containing the RE were mounted on gelatin-chromalum-coated slides and stained with neutral red and cresyl violet.

### 2.5. Statistical analysis

All data was tested for normality using the Shapiro-Wilk test. For normal data, the experiments testing the impact of stress on the RE recordings were analyzed using two-way ANOVA, with the main factors being sex (males × females) and condition (Naïve × Stress) or age, followed by Tukey’s posthoc test. Kruskal-Wallis statistic was used for non-parametric data followed by Dunn’s multiple comparisons test. Normal data are presented as mean ± SEM in a bar plot and non-normal data were presented as a median and interquartile range in a violin plot. *p* < 0.05 was defined as significant. The mean and ± SEM and percentage values for all groups are presented in the Supplementary Tables 1 and 2.

## 3. Results

### 3.1. Early adolescence stress did not alter the number of spontaneously active RE neurons after 1–2 and 5–6 weeks in either sex

**Fig. 2.**
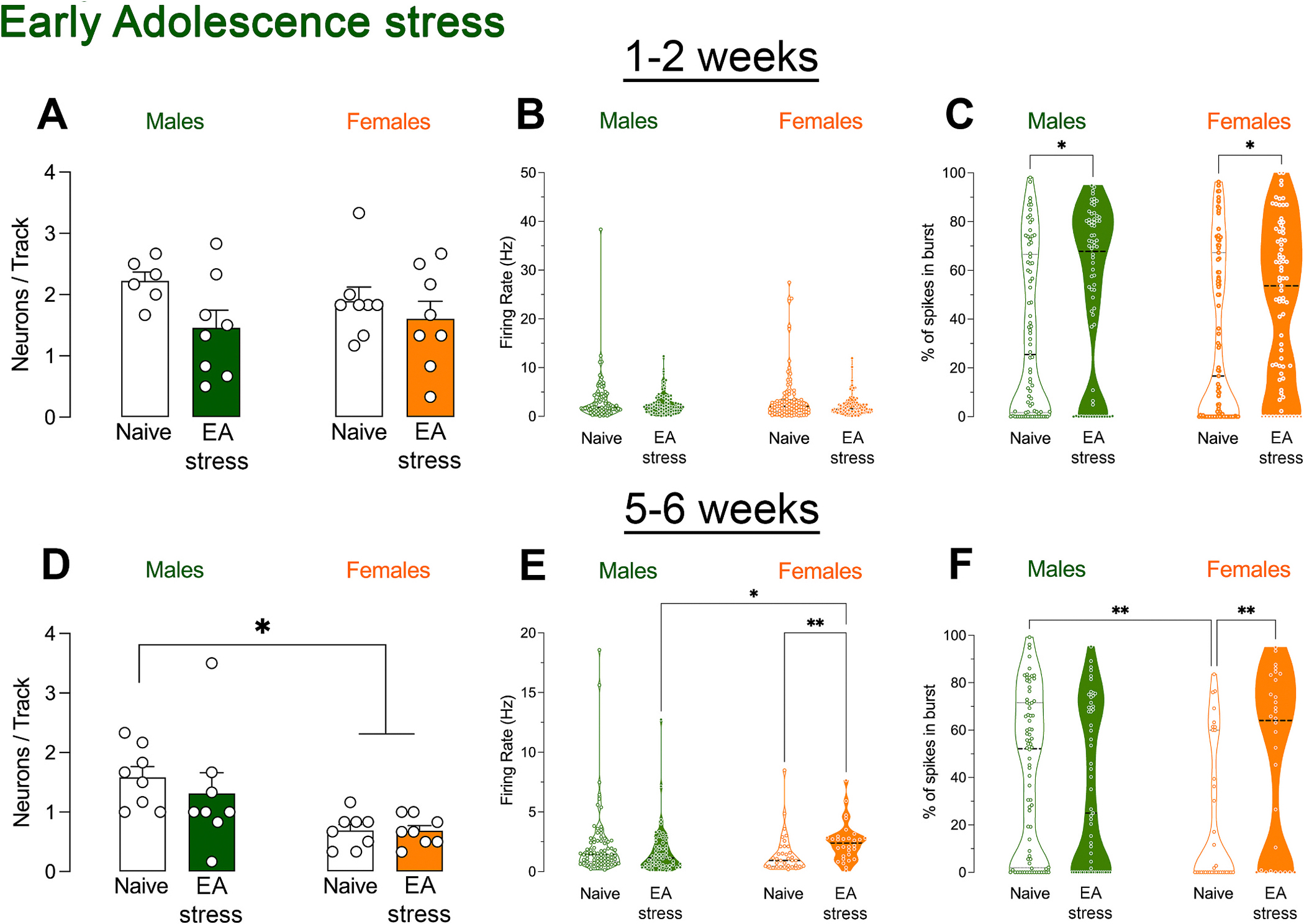
Early adolescence stress induces a long-term increase in the percentage of spikes in bursts in RE neurons of females. Short-term (1–2 weeks) analysis of the impact of early adolescence stress showed no changes in the number of RE neurons per track (A) or the firing rate (B) in males and females. However, early adolescence stress increased the percentage of spikes in bursts in both males and females after 1–2 weeks (C). In the long term (5–6 weeks), early adolescence stress did not affect the number of RE neurons per track, but a sex effect was observed, with females having fewer neurons per track than males (D). Additionally, early adolescence stress increased the firing rate of RE neurons (E) and the percentage of spikes in bursts in females after 5–6 weeks (F). *p < *0.05 Tukey’s multiple comparison post hoc*. EA: early adolescence.

Early adolescence stress did not affect the number of spontaneous active principal neurons per track of male and females rats after 1–2 weeks (*p* > 0.05, Kruskal-Wallis, Fig. 2A). The neuron firing rates were not affected by early adolescence stress in either sex (p > 0.05, Kruskal-Wallis, Fig. 2B). The % of spikes in bursts was increased in males and females exposed to early adolescence stress (H = 16.49, *p* < 0.05, Kruskal-Wallis, Fig. 2C).

RE recording 5–6 weeks post-early adolescence stress did not show a significant effect of stress on the number of spontaneously active principal neurons per track in male and female rats (*p* > 0.05, Kruskal-Wallis, Fig. 2D). However, there was observed a sex effect on the number of neurons per track, with naïve and stressed females having fewer active neurons per track than naïve and stressed males (H = 1546, *p* < 0.05, Kruskal-Wallis, Fig. 2D). Early adolescence stress increased the RE neuron firing rate of females without affecting males after 5–6 weeks (H = 13.49, p < 0,05, Kruskal-Wallis, Fig. 2E). The % of spikes in bursts was higher in females exposed to early adolescence stress compared to naïve condition (H = 13.64, *p* < 0.05 Kruskal-Wallis, Fig. 2F). Additionally, the % of spikes in bursts in naïve males was higher than in naïve females (p < 0.05, Fig. 2F).

### 3.2. Late adolescence stress increased the number of spontaneously active RE neurons after 1–2 and 5–6 weeks only in females

**Fig. 3.**
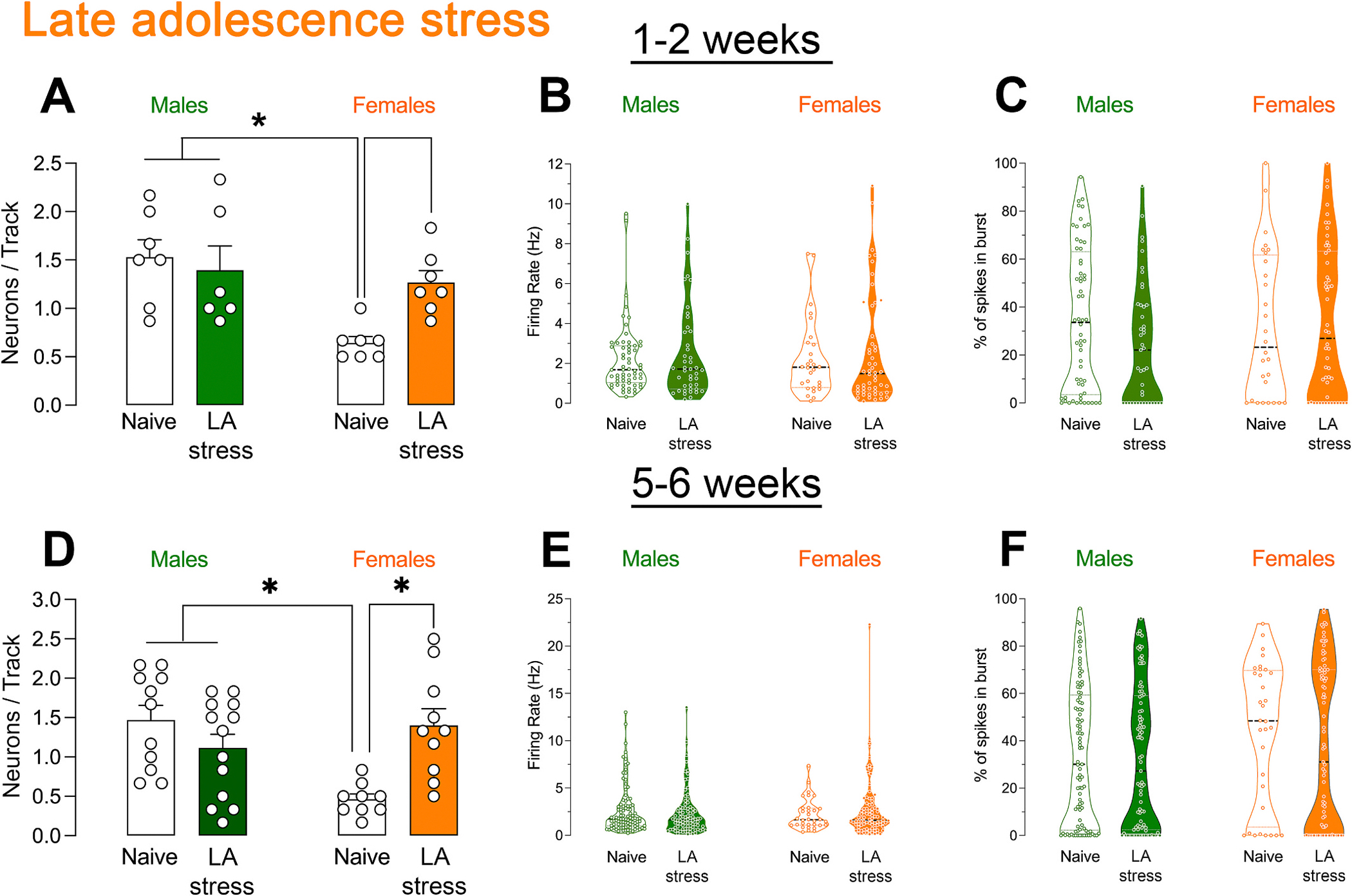
Late adolescence stress increases the number of RE neurons per track in females both in the short- and long-term. The number of RE neurons per track was increased exclusively in females 1–2 weeks after late adolescence stress (A), while no significant effects were observed on firing rate (B) or the percentage of spikes in bursts (C). At 5–6 weeks after late adolescence stress, the number of RE neurons per track remained elevated only in females (D), with no changes in neuronal firing rate (E) or the percentage of spikes in bursts (F). A sex difference was observed, with females exhibiting fewer RE neurons per track than males (A and D). *p < *0.05, Tukey’s multiple comparison post hoc test*. LA: late adolescence.

Late adolescence stress increased the number of spontaneously active neurons per track in females (H = 13.60, *p* < 0.05, Kruskal-Wallis; Fig. 3A) but not males (*p* > 0.05, Fig. 3A) after 1–2 weeks. Indeed, naïve female rats had fewer active neurons per track than naïve males (*p* < 0.05, Kruskal-Wallis; Fig. 3A). Late adolescence stress did not alter the neuron firing rate and % of spikes in bursts across sex and condition after 1–2 weeks (*p* > 0.05, Kruskal-Wallis; Fig. 3B and C).

Similar to 1–2 weeks, late adolescence stress increased the number of spontaneously active neurons per track in the RE after 5–6 weeks in females (Stress-Sex interaction, F_1,39_ = 13.61, *p* < 0.05; Stress, *p* > 0.05; Two-way ANOVA; Fig. 3D) but not males (*p* > 0.05, Two-way ANOVA; Fig. 3D). Naïve females also had fewer active neurons per track than naïve males (p < 0.05, Kruskal-Wallis; Fig. 3D). Late adolescence stress did not alter the neuron firing rate and % of spikes in bursts across sex and condition after 5–6 weeks (*p* > 0.05, Kruskal-Wallis; Fig. 3E and F).

### 3.3. Early adolescence stress increases the proportion of RE neurons burst-firing in females

**Fig. 4.**
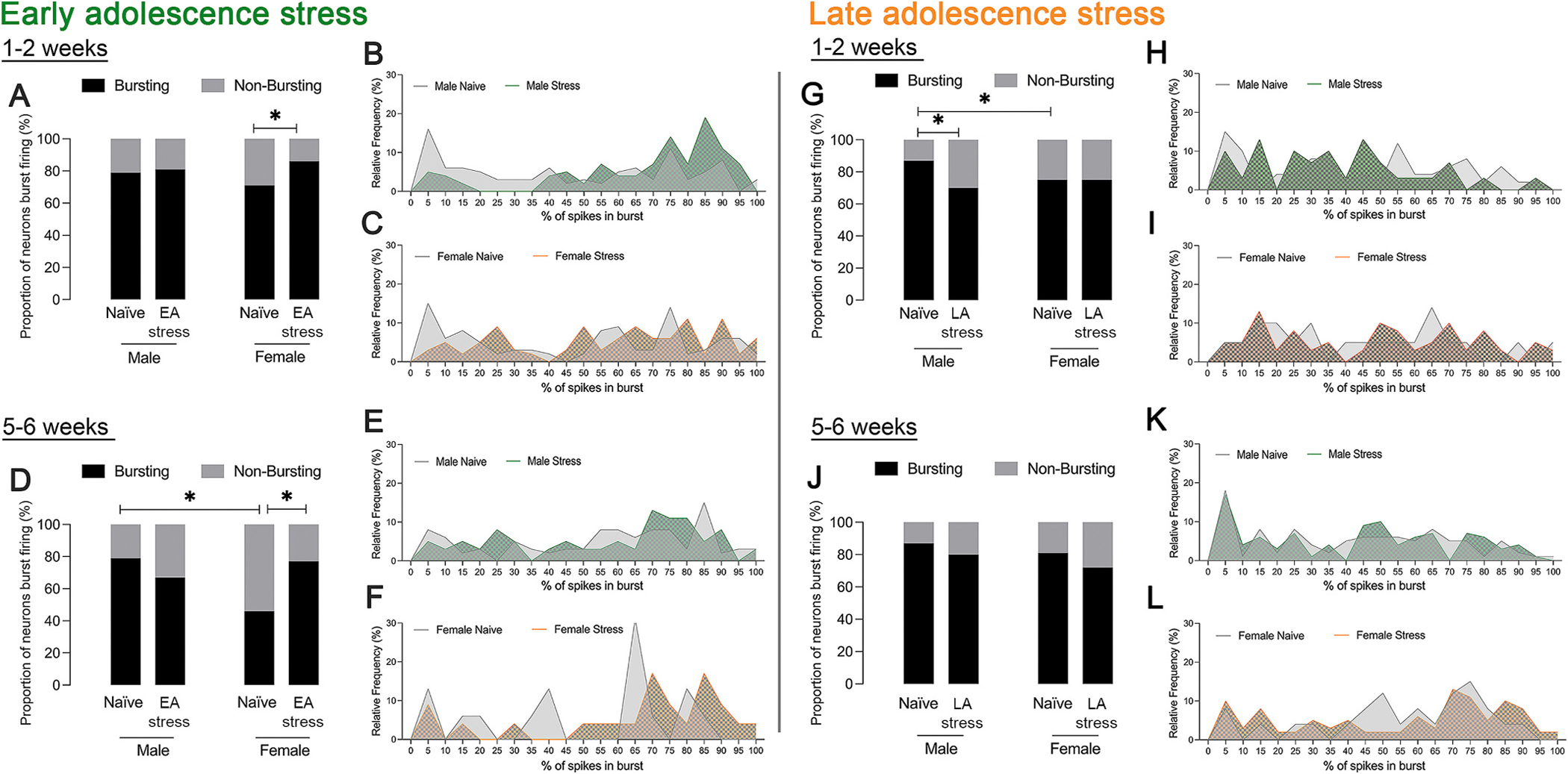
Early adolescence stress induces a sustained increase in the proportion of RE neurons firing in bursts in females. Early adolescence stress increased the proportion of RE neurons firing in bursts in females after 1–2 weeks (A) but did not alter the distribution of the percentage of spikes in bursts among burst-firing neurons in males (B) or females (C). At 5–6 weeks after early adolescence stress, stressed females continued to show a higher proportion of RE neurons firing in bursts (D), with no changes in the distribution of the percentage of spikes in bursts among burst-firing neurons in males (E) or females (F). Late adolescence stress increased the proportion of RE neurons firing in bursts in males after 1–2 weeks (G) but did not alter the distribution of the percentage of spikes in bursts among burst-firing neurons in males (H) or females (I). Late adolescence stress did not lead to long-term changes in the proportion of RE neurons exhibiting burst-firing properties after 5–6 weeks (J). No changes were observed in the distribution of the percentage of spikes in bursts among burst-firing neurons in males (K) or females (L) after 5–6 weeks *p < *0.05, Chi-square test*. EA: early adolescence; LA: late adolescence.

**Fig. 5.**
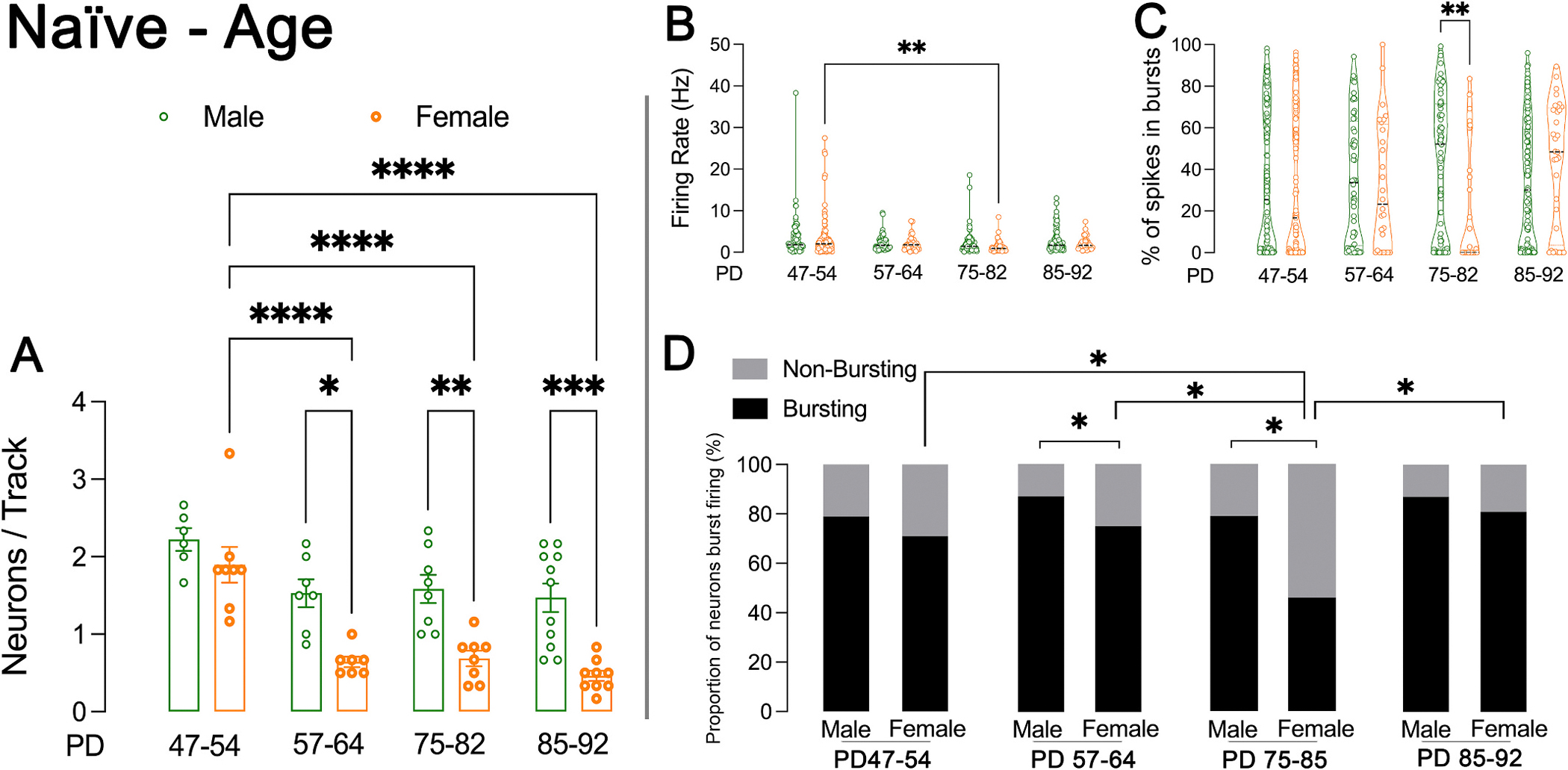
Neurodevelopmental changes of RE activity of naïve male and females. Naïve females between PD47–54 had a greater number of spontaneously active RE neurons per track compared to older females (starting from PD57) and were not different from age-matched naïve males. In contrast, older females (>PD57) exhibited a lower number of RE neurons per track compared to their age-matched naïve males (A). Females recorded between PD47–54 showed a higher firing rate compared to adult females (PD75–82) (B). A reduction in the percentage of spikes in bursts was observed in naïve females recorded during PD75–82 compared to age-matched males (C). The proportion of RE neurons firing in bursts decreases during early adulthood in females (PD57–64 and PD75–85) which also have lower levels than those observed in age-matched males (D).*p < *0.05, Tukey’s multiple comparison post hoc and Chi-square test*.

Females subjected to early adolescence stress have a higher proportion of RE neurons with burst-firing properties after 1–2 weeks (Z = 2.58, Chi-Square, p < 0.05; Fig. 4A) and 5–6 weeks (Z = 4.51, Chi-Square, p < 0.05; Fig. 4D) after stress. The proportion of RE neurons showing burst-firing properties did not change in males for both time points (Chi-Square, *p* > 0.05, Fig. 4A and D). However, naïve males had a higher proportion of RE neurons showing burst-firing than naïve females at PD75–82 (5–6 weeks time-point; Z = 4.82, Chi-Square, *p* < 0.05; Fig. 4D). The distribution of RE neurons burst-firing did not differ across stressed female and males after 1–2 weeks (Kolmogorov – Smirnov, *p* > 0.05, Fig. 4B and C) and 5–6 weeks of early adolescence stress (Kolmogorov – Smirnov, p > 0.05, Fig. 4E and F).

Late adolescence stress increased the proportion of neurons burst-firing in males after 1–2 weeks (Z = 2.93, Chi-Square, *p* < 0.05; Fig. 4G) but not 5–6 weeks (Chi-Square, p > 0.05; Fig. 4J). At 1–2 weeks timepoint, naïve males had a higher proportion of RE neurons burst-firing than naïve females (Z = 2.16, Chi-Square, *p* < 0.05; Fig. 4G). The distribution of RE neurons burst firing did not differ across stressed female and males after 1–2 weeks (Kolmogorov – Smirnov, *p* > 0.05, Fig. 4H and I) and 5–6 weeks of late adolescence stress (Kolmogorov – Smirnov, p > 0.05, Fig. 4K and L).

The relative frequency (%) analysis of firing rate did not change across the groups for any timepoint after early adolescence stress (Kolmogorov – Smirnov, p > 0.05; Supplementary Fig. 1A–D) and late adolescence Stress (Kolmogorov – Smirnov, p > 0.05; Supplementary Fig. 2A–D).

A significant degree of correlation was found for firing rate and % of spikes in bursts for most of the groups in that RE neurons with higher firing rates showed a lower percentage of spikes in burst (Supplementary Fig. 3A–E and I–P). The only groups that did not demonstrate a significant correlation between these measures were Male-Early Stress, Female-Naïve, and Female-Early Stress after 5–6 weeks of early adolescence stress (*p* > 0.05, Supplementary Fig. 3F, G, and H).

### 3.4. Females have a lower number of spontaneously active RE neurons than males in early adulthood and later

Analysis of the naïve male and female rats of different ages was conducted to evaluate if there is a sex-specific developmental effect on RE activity. Age and sex effect was found but not their interaction (Age, F_3,56_ = 17.92, *p* < 0.05; Sex, F_1,56_ = 45.61, p < 0.05; Interaction, p > 0.05; Two-Way ANOVA; Fig. 5A). At PD47–54, female and male rats did not differ in the number of spontaneously active neurons per track (p > 0.05, Fig. 5A). However, at a later age, naïve female rats had fewer active neurons per track compared to naïve males (PD57–64, PD75–82, PD85–92, p < 0.05 male × female; Fig. 5A). Indeed, comparations within the same sex at different ages demonstrated that females at PD47–54 had a higher number of spontaneously active neurons per track than older females (PD57–64, PD75–82, PD85–92, p < 0.05; Fig. 5A) which demonstrates a developmental effect on RE activity only in females.

Analysis of the firing rate of RE neurons showed that naïve females at PD47–54 had a higher firing rate than naïve females at P75–82 (H = 20.66, p < 0.05, Kruskal-Wallis; Fig. 5B) but not when compared to PD57–64 and PD85–92 (p > 0.05). No difference was observed across different ages in naïve males (p > 0.05) nor males and females in comparison within the same age (p > 0.05). Additionally, the analysis of % of spikes in bursts of RE neurons only revealed a difference between male and female rats at PD75–82 (H = 15.74, p < 0.05; Fig. 5C) with the female having a lower % of spikes in bursts. No other difference was detected in the multiple comparison test.

The proportion of RE neurons burst-firing differs across different age periods only in females (Fig. 5D), with females at PD75–82 having fewer burst firing RE neurons compared to PD47–54 (Z = 3.59, p < 0.05, Chi-square test), PD57–64 (Z = 4.19, p < 0.05, Chi-square test), and PD85–92 (Z = 5.14, p < 0.05, Chi-square test).

The relative frequency (%) analysis of firing rate did not indicate changes in the distribution of the RE neurons across all ages (Supplementary Fig. 4A–D). The % of spikes in bursts distribution was different for PD 75–82 between naïve male and females (D = 0.48, p < 0.05, Kolmogorov – Smirnov; Supplementary Fig. 5C) but not for PD47–54, PD57–64, PD85–92 (p > 0.05, Kolmogorov – Smirnov; Supplementary Fig. 5A, B, and D).

## 4. Discussion

The present study demonstrates that early and late adolescence stress selectively impact RE electrophysiological activity in a sex-specific manner. Early adolescence stress increases the proportion of RE neurons firing in bursts in both sexes, whereas late adolescence stress leads to a higher number of active RE neurons per track only in females. In contrast, RE activity in males is not strongly affected by either stress condition. These findings suggest that stress-induced alterations in RE activity may represent a critical neurobiological mechanism underlying vulnerability to neuropathological states of females.

Females were affected by early and late adolescence stress, but with distinct electrophsyiological outcomes. While early adolescence stress induced a long-term increase in burst firing activity, late adolescence stress led to a sustained increase in the number of spontaneously active RE neurons per track. This difference may be due to the influence of distinct upstream regions regulating RE activity (Vertes, 2002; Vertes et al., 2007). Previous studies have shown that the thalamic reticular nucleus (TRN) regulates the number of active neurons per track in the RE, while burst activity is influenced by direct inputs from the infralimbic prefrontal cortex (ilPFC) to the RE (Zimmerman and Grace, 2018). One possibility is that the activity of ilPFC and TRN is affected by stressors during critical periods of neurodevelopment that would impact RE function. Recent evidence suggests that the neurodevelopment of parvalbumin (PV) interneurons in the TRN occurs later in females (Landzhov et al., 2004). Males exhibit higher TRN PV immunoreactivity than females at PD 20 (Landzhov et al., 2004). By PD28, males also show a greater PV cell density in the TRN compared to age-matched females; however, by PD42, PV cell density levels appear similar between the sexes (Pirone et al., 2020). This delayed maturation may render the TRN more vulnerable to stress-induced PV damage particularly in females exposed to late adolescence stress. Therefore, stress may impair PV interneurons in the TRN and consequently disrupting inhibitory inputs from the TRN to the RE that could lead to disinhibition of the RE which leads to the increased number of spontaneously active neurons per track. However, this idea is still speculative, and future studies need to further explore the interplay of TRN and RE in regulating stress vulnerability.

The RE serves as a crucial relay for vHip regulation (Griffin, 2015; Vertes et al., 2007). This aligns with the hypothesis that heightened RE activity would drive vHip hyperexcitability following late adolescence stress exclusively in females, as reported in our previous studies (Zhu and Grace, 2022). Hyperexcitability in the limbic hippocampus is proposed to be a key pathological feature of schizophrenia that underlies symptoms through multiple projections (Grace, 2016; Grace and Uliana, 2023; Wegrzyn et al., 2022). The vHip pathway, via the nucleus accumbens and ventral pallidum, is known to regulate dopaminergic activity in the VTA (Lodge and Grace, 2006). Notably, the effects of late adolescence stress on vHip hyperexcitability and hyperdopaminergia in the VTA appear to be sex-specific, occurring only in females (Zhu and Grace, 2022). We propose that increased vHip activity contributes to the hyperdopaminergic state in females exposed to late adolescent stress, with the RE playing a pivotal role in driving vHip activity. Future studies employing a more direct approach will elucidate the role of RE regulation in potentially regulating dopaminergic activity in vHip.

The persistent increase in burst firing activity observed in females after early adolescence stress may be driven by the ilPFC, as this region undergoes extensive maturation during this period, particularly in terms of inhibitory neurotransmission (Caballero et al., 2016; Le Magueresse and Monyer, 2013). Studies demonstrate that PFC PV interneurons increase throughout the juvenile and adolescent periods in rats (Caballero et al., 2014). In both males and females, there is a marked increase in PV+ interneurons in the ilPFC from PD20 to PD40, followed by a later decrease at PD70 (Gildawie et al., 2020). Furthermore, data suggest that PV maturation in the PFC of females is regulated by estradiol (Piekarski et al., 2017; Wu et al., 2014). Indeed, the formation of the perineuronal extracellular matrix, which is crucial for PV stabilization, is mediated by puberty onset in females around PD35 but not in males (Drzewiecki et al., 2020; Takesian and Hensch, 2013). This process may be associated with the closure of critical periods of neuroplasticity and PV stabilization. As females usually present an earlier puberty onset (around PD30–40) compared to males (PD42–55) (Bell, 2018; Ojeda et al., 1980), early adolescence stress occuring during PD31–40 puberty period may disrupt the neurodevelopmental trajectories of PV ilPFC maturation which in turn may affect the bursting-firing properties of RE neurons through their direct ilPFC projections in females. In males, only a short-term increase was observed after early adolescence stress, which could suggest that the later onset of puberty in males may normalize the effects of stress during prepuberty. Moreover, it has been reported that direct inhibition of projections from the ilPFC to the RE increases burst firing (Zimmerman and Grace, 2018), suggesting that ilPFC dysregulation could drive RE neurons to fire in bursts. Moreover, a recent study demonstrated that RE projections to GABAergic interneurons in the ilPFC modulate pain responses and motivational behaviors (Bao et al., 2025). Based on these findings, we propose that footshock during stress procedures could, in part, activate the RE by inducing some degree of pain perception, which thereby contributes to the anxiety state. This effect, especially during early adolescence, may increase the excitatory drive from the RE to the ilPFC. Thus, it may lead to a dysregulated maturation and activity of ilPFC; in particular of females undergoing puberty. Such changes could later result in ilPFC dysregulation and altered connectivity back to the RE, as the ilPFC appears to be the primary driver of RE neuronal bursts. Our findings in females exposed to early adolescence stress shows an increased proportion of RE neurons firing in bursts, which is observed both in the short term (1–2 weeks) and long term (5–6 weeks) following stress. Deficits in ilPFC glutamatergic projections to the RE could lead to hyperpolarization of RE neurons, promoting greater de-inactivation of T-type calcium channels and resulting in larger low-threshold spikes (Gutierrez et al., 2001; Sherman, 2001a).

One limitation of this study is that the animals were under anesthesia, which may affect cellular activity. Anesthesia has been shown to influence the firing properties of thalamic regions, including burst firing (Alkire et al., 2008). Additionally, bursting activity is known to increase during both sleep and anesthesia states (Bezdudnaya et al., 2006; Sherman, 2001a, 2001b). However, since all animals were under the same conditions, the observed increase in bursting and RE neurons per track remains a relevant measure and may give insights to disrupted inputs. Burst firing in thalamic regions has been documented across different species and levels of consciousness, including both sleep and wakefulness (Jeanmonod et al., 1996; Kim et al., 2009; Nicolelis and Fanselow, 2002; Ramcharan et al., 2005; Swadlow and Gusev, 2001). This suggests that the burst firing data could provide meaningful evidence applicable across species. While baseline activity is probably affected by anesthesia, it should not qualitatively alter the condition effect and brain connectivity. The RE, as a higher-order thalamic relay, exhibits pronounced burst activity which suggests that this firing pattern may have functional significance in this region (Ramcharan et al., 2005; Wei et al., 2011), particularly in the context of neuropathological conditions. In addition, it is possible that the depth of anesthesia could influence the activity parameters of RE. However, while this may result in a quantitative difference from awake animals, our group previously evaluated RE recordings under the same conditions and found results similar to those presented here (Zimmerman and Grace, 2018) which demonstrates consistent outcomes across different experimenters. In particular, the number of active RE neurons per track in males (average of 1.5 neurons per track) (Zimmerman and Grace, 2018) is consistent with the male naïve data in adult rats shown here (1.58 neurons/track PD75–82 and 1.47 neurons/track PD85–92). While the influence of depth of anesthesia cannot be entirely ruled out, the consistent results and low variability in the presented study suggest that the statistical values are not likely affected by anesthesia. Moreover, the naïve groups show consistent variability within groups, whereas the stress groups display greater variability across age and sex. This may reflect individual differences in response to stress. Future studies aim to better characterize the electrophysiological profile of RE activity and it correlation with connectivity with downstream regions, and the behavioral consequences of these patterns under stress condition.

In males, neither early nor late adolescence stress significantly affects RE activity. Only a short-term (1–2 weeks) increase in the proportion of neurons firing in bursts was observed following late adolescence stress. This aligns with the proposition that the neurobiological circuits underlying stress vulnerability are sex-specific, with females being affected by late adolescence stress via RE and males being affected by early adolescence stress via BLA. Previous studies have shown that males exposed to early adolescence (PD21–30) stress exhibit increased BLA activity in both the short- and long-term (Uliana et al., 2021; Zhu and Grace, 2022). It has been proposed that BLA hyperexcitability drives increased vHip activity through PV disruption in the vHip (Gomes et al., 2019b; Zhu and Grace, 2022). This would underlie the susceptibility to heightened dopaminergic activity in the VTA and behavioral dysregulation (Gomes and Grace, 2017; Zhu and Grace, 2022) which is not observed in females subjected to early adolescence stress (Klinger et al., 2019; Zhu and Grace, 2022). In humans, peripubertal adversity is associated with increased amygdala volume (Pechtel et al., 2014), which aligns with findings that early adolescence stress in males lead to BLA-dependent VTA-associative striatal hyperactivity (Gomes and Grace, 2017; Zhu and Grace, 2022). DA hyperactivity in the associative striatum is a core neurobiological dysfunction contributing to psychosis in humans (Kegeles et al., 2010; Laruelle, 1998; Laruelle and Abi-Dargham, 1999).

On the other hand, late adolescence stress susceptibility in females is associated with medial VTA mesolimbic hyperactivity (Zhu and Grace, 2022) which would also align with affective disorders (Grace, 2016). Notably, RE dysfunction also appears to be implicated in affective disorders (Goveas et al., 2011; Sheline et al., 2009). Preclinical studies indicate that RE lesion and inactivation mitigate the negative motivational effects induced by adult stress models (Kafetzopoulos et al., 2018; Uliana et al., 2022), along with the restoration of dopaminergic function following RE inactivation (Uliana et al., 2022). In addition, the role of RE in affective responses during adulthood appears to be non-sex specific (Kafetzopoulos et al., 2025). This suggests that the RE may also represent a neurobiological node of susceptibility for affective disorders, with females likely being more susceptible during late adolescent neurodevelopmental state. In fact, both young adult and adult females exhibit a lower number of RE neurons per track compared to males that may render them more vulnerable to stressors that drive RE hyperactivity.

While we postulate that males may be more susceptible to the long-term effects of stress experienced during early adolescent periods and females during late adolescent periods, different studies also demonstrate that females can be susceptible to stress earlier on. For example, females exposed to peri-puberty stress demonstrate a decreased prepulse inhibition, which males also demonstrate but in a lesser magnitude. Also, post-pubertal stress in males and females did not produce an effect on anxiety, prepulse inhibition response, and despair behavior (Drzewiecki et al., 2021). This differs from our concept of stress susceptibility. While we propose that specific behaviors, such as anxiety and recognition memory, can indicate stress susceptibility, we do not exclude other behavioral indicators that may also be affected by stress. Further studies are required to expand our understanding of different stress response domains. However, we speculate that the type of stress and frequency of stress may be a driver for the difference in this study as it relates to the previous studies from our group (Gomes et al., 2019a; Gomes and Grace, 2017; Klinger et al., 2019). Prepulse inhibition may be a more sensitive measure for detecting the early onset of disrupted circuit under milder stress conditions. As stress intensity increases, the disruption may extend beyond sensorimotor gating to also impair cognitive functions, heighten anxiety-like behaviors, and alter locomotor activity.

In the broader discussion of sex-dependent responses in the RE, the current literature remains limited. However, some evidence suggests that c-Fos expression in the RE is lower in females relative to males following acute stress exposure during adulthood (Kafetzopoulos et al., 2025; Kim and Chung, 2021). This contrasts with our findings in the RE, which indicate that late adolescent stress increases the number of active projection neurons in females, while early adolescent stress drives RE neurons to burst-fire. We attribute these differences to both the developmental timing of stress exposure and its repeated nature, which may lead to greater disruptions in the RE and its network, potentially impacting neurobiological domains relevant to schizophrenia. For instance, a study demonstrated that administration of MK-801, an NMDA receptor antagonist commonly used to model certain aspects of schizophrenia, increased c-Fos expression in the RE of both sexes, but with a greater magnitude in females (D’Souza et al., 1999). This aligns with our hypothesis that the hyperdopaminergic state observed in females exposed to late adolescent stress may be due to increased RE activation. Additional evidence points to other sex-specific mechanisms in the RE. For example, females have a greater number of mast cells in the RE, suggesting that stress vulnerability in females may be mediated by enhanced inflammatory processes (Goldschmidt et al., 1984). Moreover, structural differences have also been described, with females exhibiting longer axonal terminals of PFC projections to the RE which may indicate a greater PFC–RE connectivity in females (Smith et al., 2022). Additionally, females were reported to display less despair-like behavior and greater c-Fos expression in the mPFC compared to males following a prolonged forced swim test (Colom-Lapetina et al., 2017) that could align with a greater mPFC activity in females and suggests a possible connection with the RE. However, these details remain speculative as the field continues to explore the gaps in differences between males and females. Nonetheless, it is proposed that the RE may either serve as the primary driver of sex-specific behavioral response or function as a sex-dependent mechanism that enables males and females to achieve similar behavioral responses through distinct pathways (Cassel et al., 2025). Consistent with this idea, our study supports the notion that adolescent stress can induce a long-term hyperdopaminergic state in both sexes (Gomes and Grace, 2017; Zhu and Grace, 2022), with the key differences residing in the timing of stress exposure and the underlying neurobiological circuits. Males appear to be primarily driven by amygdala activity (Uliana et al., 2021; Zhu and Grace, 2022), while females may be more reliant on RE mechanisms demonstrated in the present study.

Another interesting finding from our study is that females do not have a decrease in RE activity during PD47–54. This could reflect a distinct neurodevelopmental trajectory of RE maturation in females which likely is influenced by TRN activity. As mentioned previously, PV maturation in the TRN appears to occur later in females (Landzhov et al., 2004; Pirone et al., 2020), which aligns with the idea that RE may not yet be receiving fully mature inhibitory inputs from the TRN. This lack of mature inhibition could lead to RE disinhibition, resulting in increased RE activity observed during PD47–54 in females. Starting from PD57, females had lower numbers of active principal RE neurons per track compared to males and to earlier female age, suggesting that RE and TRN may be achieving full maturation in females during early adulthood. However, it is unclear whether males undergo a similar process at an earlier developmental stage than the one recorded in this study.

In conclusion, our findings demonstrate sex- and age-specific effects of stress on RE activity, with females exhibiting distinct electrophysiological outcomes in response to early and late adolescence stress while males were not affected. These results suggest that RE dysfunction may serve as a critical mediator of stress susceptibility in females which could be influencing vHip hyperexcitability and mesolimbic dopaminergic dysregulation. The possible role of TRN maturation in shaping RE activity highlights a developmental window of vulnerability in females. Our findings reinforce the idea that stress-induced alterations in limbic-thalamic circuits may contribute to neuropsychiatric disorders in a sex-dependent manner.

## Supplementary Material

1

## Appendix A. Supplementary data

Supplementary data to this article can be found online at https://doi.org/10.1016/j.schres.2025.09.018.

## References

[R1] Alkire MT, Hudetz AG, Tononi G, 2008. Consciousness and anesthesia. Science 322, 876–880. 10.1126/science.1149213. 18988836 PMC2743249

[R2] Atrooz F, Alkadhi KA, Salim S, 2021. Understanding stress: insights from rodent models. Curr Res Neurobiol 2, 100013. 10.1016/j.crneur.2021.100013. 36246514 PMC9559100

[R3] Bao S-T, Rao F, Yin C, Niu Y, Cao J-L, Xiao C, Zhou C, 2025. Excitatory projections from the nucleus reuniens to the medial prefrontal cortex modulate pain and depression-like behaviors in mice. PLoS Biol. 23, e3003170. 10.1371/journal.pbio.3003170. 40392890 PMC12091829

[R4] Bell MR, 2018. Comparing postnatal development of gonadal hormones and associated social behaviors in rats, mice, and humans. Endocrinology 159, 2596–2613. 10.1210/en.2018-00220. 29767714 PMC6692888

[R5] Bezdudnaya T, Cano M, Bereshpolova Y, Stoelzel CR, Alonso J-M, Swadlow HA, 2006. Thalamic burst mode and inattention in the awake LGNd. Neuron 49, 421–432. 10.1016/j.neuron.2006.01.010. 16446145

[R6] Caballero A, Thomases DR, Flores-Barrera E, Cass DK, Tseng KY, 2014. Emergence of GABAergic-dependent regulation of input-specific plasticity in the adult rat prefrontal cortex during adolescence. Psychopharmacology 231, 1789–1796. 10.1007/s00213-013-3216-4. 23907651 PMC3873346

[R7] Caballero A, Granberg R, Tseng KY, 2016. Mechanisms contributing to prefrontal cortex maturation during adolescence. Neurosci. Biobehav. Rev. 70, 4–12. 10.1016/j.neubiorev.2016.05.013. 27235076 PMC5074870

[R8] Cassel J-C, Panzer E, Guimaraes-Olmo I, Cosquer B, Pereira De Vasconcelos A, Stephan A, 2025. Is there something sexual in the ventral midline thalamus? Brain Struct. Funct. 230, 26. 10.1007/s00429-024-02869-2. 39760747

[R9] Colom-Lapetina J, Begley SL, Johnson ME, Bean KJ, Kuwamoto WN, Shansky RM, 2017. Strain-dependent sex differences in a long-term forced swim paradigm. Behav. Neurosci. 131, 428–436. 10.1037/bne0000215. 28805432

[R10] Croft J, Heron J, Teufel C, Cannon M, Wolke D, Thompson A, Houtepen L, Zammit S, 2019. Association of trauma type, age of exposure, and frequency in childhood and adolescence with psychotic experiences in early adulthood. JAMA Psychiatry 76, 79–86. 10.1001/jamapsychiatry.2018.3155. 30477014 PMC6490231

[R11] Croft J, Martin D, Madley-Dowd P, Strelchuk D, Davies J, Heron J, Teufel C, Zammit S, 2021. Childhood trauma and cognitive biases associated with psychosis: a systematic review and meta-analysis. PloS One 16, e0246948. 10.1371/journal.pone.0246948. 33630859 PMC7906349

[R12] Daviu N, Molina P, Nadal R, Belda X, Serrano S, Armario A, 2024. Influence of footshock number and intensity on the behavioral and endocrine response to fear conditioning and cognitive fear generalization in male rats. Prog. Neuropsychopharmacol. Biol. Psychiatry 135, 111112. 10.1016/j.pnpbp.2024.111112. 39094926

[R13] Di Prisco GV, Vertes RP, 2006. Excitatory actions of the ventral midline thalamus (rhomboid/reuniens) on the medial prefrontal cortex in the rat. Synapse 60, 45–55. 10.1002/syn.20271. 16596625

[R14] Dolleman-van der Weel MJ, Witter MP, 2020. The thalamic midline nucleus reuniens: potential relevance for schizophrenia and epilepsy. Neurosci. Biobehav. Rev. 119, 422–439. 10.1016/j.neubiorev.2020.09.033. 33031816

[R15] Dolleman-van der Weel MJ, Griffin AL, Ito HT, Shapiro ML, Witter MP, Vertes RP, Allen TA, 2019. The nucleus reuniens of the thalamus sits at the nexus of a hippocampus and medial prefrontal cortex circuit enabling memory and behavior. Learn. Mem. 26, 191–205. 10.1101/lm.048389.118. 31209114 PMC6581009

[R16] Drzewiecki CM, Willing J, Juraska JM, 2020. Influences of age and pubertal status on number and intensity of perineuronal nets in the rat medial prefrontal cortex. Brain Struct. Funct. 225, 2495–2507. 10.1007/s00429-020-02137-z. 32914251 PMC7554170

[R17] Drzewiecki CM, Willing J, Cortes LR, Juraska JM, 2021. Adolescent stress during, but not after, pubertal onset impairs indices of prepulse inhibition in adult rats. Dev. Psychobiol. 63, 837–850. 10.1002/dev.22111. 33629385 PMC8355031

[R18] D’Souza DN, Harlan RE, Garcia MM, 1999. Sexual dimorphism in the response to N-methyl-d-aspartate receptor antagonists and morphine on behavior and c-Fos induction in the rat brain. Neuroscience 93, 1539–1547. 10.1016/S0306-4522(99)00229-8. 10501478

[R19] Duan AR, Varela C, Zhang Y, Shen Y, Xiong L, Wilson MA, Lisman J, 2015. Delta frequency optogenetic stimulation of the thalamic nucleus reuniens is sufficient to produce working memory deficits: relevance to schizophrenia. Biol. Psychiatry 77, 1098–1107. 10.1016/j.biopsych.2015.01.020. 25891221 PMC4444380

[R20] Gildawie KR, Honeycutt JA, Brenhouse HC, 2020. Region-specific effects of maternal separation on perineuronal net and parvalbumin-expressing interneuron formation in male and female rats. Neuroscience 428, 23–37. 10.1016/j.neuroscience.2019.12.010. 31887358

[R21] Godsil BP, Kiss JP, Spedding M, Jay TM, 2013. The hippocampal–prefrontal pathway: the weak link in psychiatric disorders? Eur. Neuropsychopharmacol. 23, 1165–1181. 10.1016/j.euroneuro.2012.10.018. 23332457

[R22] Goldschmidt RC, Hough LB, Glick SD, Padawer J, 1984. Mast cells in rat thalamus: nuclear localization, sex difference and left-right asymmetry. Brain Res. 323, 209–217. 10.1016/0006-8993(84)90291-9. 6084538

[R23] Gomes FV, Grace AA, 2017. Prefrontal cortex dysfunction increases susceptibility to schizophrenia-like changes induced by adolescent stress exposure. Schizophr. Bull. 43, 592–600. 10.1093/schbul/sbw156. 28003467 PMC5464092

[R24] Gomes FV, Zhu X, Grace AA, 2019a. The pathophysiological impact of stress on the dopamine system is dependent on the state of the critical period of vulnerability. Mol. Psychiatry. 10.1038/s41380-019-0514-1. PMC7056584 31488866

[R25] Gomes FV, Zhu X, Grace AA, 2019b. Stress during critical periods of development and risk for schizophrenia. Schizophr. Res. 213, 107–113. 10.1016/j.schres.2019.01.030. 30711313 PMC6667322

[R26] Goveas J, Xie C, Wu Z, Douglas Ward B, Li W, Franczak MB, Jones JL, Antuono PG, Yang Z, Li S-J, 2011. Neural correlates of the interactive relationship between memory deficits and depressive symptoms in nondemented elderly: resting fMRI study. Behav. Brain Res. 219, 205–212. 10.1016/j.bbr.2011.01.008. 21238490 PMC3062733

[R27] Grace AA, 2016. Dysregulation of the dopamine system in the pathophysiology of schizophrenia and depression. Nat. Rev. Neurosci. 17, 524–532. 10.1038/nrn.2016.57. 27256556 PMC5166560

[R28] Grace AA, Uliana DL, 2023. Insights into the mechanism of action of antipsychotic drugs derived from animal models: standard of care versus novel targets. Int. J. Mol. Sci. 24, 12374. 10.3390/ijms241512374. 37569748 PMC10418544

[R29] Griffin AL, 2015. Role of the thalamic nucleus reuniens in mediating interactions between the hippocampus and medial prefrontal cortex during spatial working memory. Front. Syst. Neurosci. 9. 10.3389/fnsys.2015.00029. PMC4354269 25805977

[R30] Gutierrez C, Cox CL, Rinzel J, Sherman SM, 2001. Dynamics of low-threshold spike activation in relay neurons of the cat lateral geniculate nucleus. J. Neurosci. 21, 1022–1032. 10.1523/JNEUROSCI.21-03-01022.2001. 11157087 PMC6762305

[R31] Herkenham M, 1978. The connections of the nucleus reuniens thalami: evidence for a direct thalamo-hippocampal pathway in the rat. J. Comp. Neurol. 177, 589–610. 10.1002/cne.901770405. 624792

[R32] Hodes GE, Epperson CN, 2019. Sex differences in vulnerability and resilience to stress across the life span. Biol. Psychiatry 86, 421–432. 10.1016/j.biopsych.2019.04.028. 31221426 PMC8630768

[R33] Jeanmonod D, Magnin M, Morel A, 1996. Low-threshold calcium spike bursts in the human thalamus. Common physiopathology for sensory, motor and limbic positive symptoms. Brain 119 (Pt 2), 363–375. 10.1093/brain/119.2.363. 8800933

[R34] Kafetzopoulos V, Kokras N, Sotiropoulos I, Oliveira JF, Leite-Almeida H, Vasalou A, Sardinha VM, Papadopoulou-Daifoti Z, Almeida OFX, Antoniou K, Sousa N, Dalla C, 2018. The nucleus reuniens: a key node in the neurocircuitry of stress and depression. Mol. Psychiatry 23, 579–586. 10.1038/mp.2017.55. 28397837 PMC5822458

[R35] Kafetzopoulos V, Kokras N, Katsaitis F, Sousa N, Leite-Almeida H, Sotiropoulos I, Dalla C, 2025. Prefrontal cortex—nucleus reuniens—hippocampus network exhibits sex-differentiated responses to stress and antidepressant treatment in rats. Psychopharmacology 242, 1627–1639. 10.1007/s00213-024-06667-w. 39162717

[R36] Kegeles LS, Abi-Dargham A, Frankle WG, Gil R, Cooper TB, Slifstein M, Hwang D-R, Huang Y, Haber SN, Laruelle M, 2010. Increased synaptic dopamine function in associative regions of the striatum in schizophrenia. Arch. Gen. Psychiatry 67, 231–239. 10.1001/archgenpsychiatry.2010.10. 20194823

[R37] Kendler KS, Karkowski LM, Prescott CA, 1999. Causal relationship between stressful life events and the onset of major depression. Am. J. Psychiatry 156, 837–841. 10.1176/ajp.156.6.837. 10360120

[R38] Kesby J, Eyles D, McGrath J, Scott J, 2018. Dopamine, psychosis and schizophrenia: the widening gap between basic and clinical neuroscience. Transl. Psychiatry 8, 30. 10.1038/s41398-017-0071-9. 29382821 PMC5802623

[R39] Kim W, Chung C, 2021. Brain-wide cellular mapping of acute stress-induced activation in male and female mice. FASEB J. 35. 10.1096/fj.202101287R. 34780680

[R40] Kim JH, Ohara S, Lenz FA, 2009. Mental arithmetic leads to multiple discrete changes from baseline in the firing patterns of human thalamic neurons. J. Neurophysiol. 101, 2107–2119. 10.1152/jn.91087.2008. 19193769 PMC2695644

[R41] Kim J, Woo J, Park Y-G, Chae S, Jo S, Choi JW, Jun HY, Yeom YI, Park SH, Kim KH, Shin H-S, Kim D, 2011. Thalamic T-type Ca2+ channels mediate frontal lobe dysfunctions caused by a hypoxia-like damage in the prefrontal cortex. J. Neurosci. 31, 4063–4073. 10.1523/JNEUROSCI.4493-10.2011. 21411648 PMC6623536

[R42] Klinger K, Gomes FV, Rincón-Cortés M, Grace AA, 2019. Female rats are resistant to the long-lasting neurobehavioral changes induced by adolescent stress exposure. Eur. Neuropsychopharmacol. 29, 1127–1137. 10.1016/j.euroneuro.2019.07.134. 31371105 PMC6773464

[R43] Landzhov B, Bozhilova-Pastirova A, Ovtscharoff W, 2004. Postnatal development of NADPH-diaphorase-reactivity and parvalbumm immunoreactivity in the auditory sector of the thalamic reticular nucleus of male and female rats. Comptes Rendus de l’Academie Bulgare des Sciences 57, 2.

[R44] Laruelle M, 1998. Imaging dopamine transmission in schizophrenia. A review and meta-analysis. Q. J. Nucl. Med. 42, 211–221. 9796369

[R45] Laruelle M, Abi-Dargham A, 1999. Dopamine as the wind of the psychotic fire: new evidence from brain imaging studies. J. Psychopharmacol. 13, 358–371. 10.1177/026988119901300405. 10667612

[R46] Le Magueresse C, Monyer H, 2013. GABAergic interneurons shape the functional maturation of the cortex. Neuron 77, 388–405. 10.1016/j.neuron.2013.01.011. 23395369

[R47] Likhtik E, Pelletier JG, Popescu AT, Paré D, 2006. Identification of basolateral amygdala projection cells and interneurons using extracellular recordings. J. Neurophysiol. 96, 3257–3265. 10.1152/jn.00577.2006. 17110739

[R48] Lodge DJ, Grace AA, 2006. The hippocampus modulates dopamine neuron responsivity by regulating the intensity of phasic neuron activation. Neuropsychopharmacology 31, 1356–1361. 10.1038/sj.npp.1300963. 16319915

[R49] Lovelock DF, Deak T, 2019. Acute stress imposed during adolescence yields heightened anxiety in Sprague Dawley rats that persists into adulthood: sex differences and potential involvement of the medial amygdala. Brain Res. 1723, 146392. 10.1016/j.brainres.2019.146392. 31446016 PMC6766421

[R50] Marshall AD, 2016. Developmental timing of trauma exposure relative to puberty and the nature of psychopathology among adolescent girls. J. Am. Acad. Child Adolesc. Psychiatry 55, 25–32 e1. 10.1016/j.jaac.2015.10.004. 26703906 PMC4691280

[R51] Nicolelis MAL, Fanselow EE, 2002. Dynamic shifting in thalamocortical processing during different behavioural states. Philos. Trans. R. Soc. Lond. B Biol. Sci. 357, 1753–1758. 10.1098/rstb.2002.1175. 12626009 PMC1693080

[R52] Ojeda SR, Andrews WW, Advis JP, White SS, 1980. Recent advances in the endocrinology of puberty. Endocr. Rev. 1, 228–257. 10.1210/edrv-1-3-228. 6112144

[R53] Pechtel P, Lyons-Ruth K, Anderson CM, Teicher MH, 2014. Sensitive periods of amygdala development: the role of maltreatment in preadolescence. Neuroimage 97, 236–244. 10.1016/j.neuroimage.2014.04.025. 24736182 PMC4258391

[R54] Piekarski DJ, Boivin JR, Wilbrecht L, 2017. Ovarian hormones organize the maturation of inhibitory neurotransmission in the frontal cortex at puberty onset in female mice. Curr. Biol. 27, 1735–1745 e3. 10.1016/j.cub.2017.05.027. 28578932 PMC5699709

[R55] Pirone A, Viaggi C, Cantile C, Giannessi E, Pardini C, Vaglini F, Miragliotta V, 2020. Morphological alterations of the reticular thalamic nucleus in Engrailed-2 knockout mice. J. Anat. 236, 883–890. 10.1111/joa.13150. 31972897 PMC7163774

[R56] Pruessner M, Iyer SN, Faridi K, Joober R, Malla AK, 2011. Stress and protective factors in individuals at ultra-high risk for psychosis, first episode psychosis and healthy controls. Schizophr. Res. 129, 29–35. 10.1016/j.schres.2011.03.022. 21497058

[R57] Rahman F, Nanu R, Schneider NA, Katz D, Lisman J, Pi H-J, 2021. Optogenetic perturbation of projections from thalamic nucleus reuniens to hippocampus disrupts spatial working memory retrieval more than encoding. Neurobiol. Learn. Mem. 179, 107396. 10.1016/j.nlm.2021.107396. 33524571 PMC7987745

[R58] Ramcharan EJ, Gnadt JW, Sherman SM, 2005. Higher-order thalamic relays burst more than first-order relays. Proc. Natl. Acad. Sci. U. S. A. 102, 12236–12241. 10.1073/pnas.0502843102. 16099832 PMC1189315

[R59] Rosenkranz JA, Grace AA, 1999. Modulation of basolateral amygdala neuronal firing and afferent drive by dopamine receptor activation in vivo. J. Neurosci. 19, 11027–11039. 10594083 10.1523/JNEUROSCI.19-24-11027.1999 PMC6784949

[R60] Sheline YI, Barch DM, Price JL, Rundle MM, Vaishnavi SN, Snyder AZ, Mintun MA, Wang S, Coalson RS, Raichle ME, 2009. The default mode network and self-referential processes in depression. Proc. Natl. Acad. Sci. U. S. A. 106, 1942–1947. 10.1073/pnas.0812686106. 19171889 PMC2631078

[R61] Sherman SM, 2001a. Tonic and burst firing: dual modes of thalamocortical relay. Trends Neurosci. 24, 122–126. 10.1016/s0166-2236(00)01714-8. 11164943

[R62] Sherman SM, 2001b. Thalamic relay functions. Prog. Brain Res. 134, 51–69. 10.1016/s0079-6123(01)34005-0. 11702563

[R63] Small SA, Schobel SA, Buxton RB, Witter MP, Barnes CA, 2011. A pathophysiological framework of hippocampal dysfunction in ageing and disease. Nat. Rev. Neurosci. 12, 585–601. 10.1038/nrn3085. 21897434 PMC3312472

[R64] Smith IF, Gursky ZH, Klintsova AY, 2022. Representation of prefrontal axonal efferents in the thalamic nucleus reuniens in a rodent model of fetal alcohol exposure during third trimester. Front. Behav. Neurosci. 16, 993601. 10.3389/fnbeh.2022.993601. 36160686 PMC9493097

[R65] Solmi M, Radua J, Olivola M, Croce E, Soardo L, Salazar De Pablo G, Il Shin J, Kirkbride JB, Jones P, Kim JH, Kim JY, Carvalho AF, Seeman MV, Correll CU, Fusar-Poli P, 2022. Age at onset of mental disorders worldwide: large-scale meta-analysis of 192 epidemiological studies. Mol. Psychiatry 27, 281–295. 10.1038/s41380-021-01161-7. 34079068 PMC8960395

[R66] Swadlow HA, Gusev AG, 2001. The impact of “bursting” thalamic impulses at a neocortical synapse. Nat. Neurosci. 4, 402–408. 10.1038/86054. 11276231

[R67] Takesian AE, Hensch TK, 2013. Balancing plasticity/stability across brain development. Prog. Brain Res. 207, 3–34. 10.1016/B978-0-444-63327-9.00001-1. 24309249

[R68] Thapar A, Riglin L, 2020. The importance of a developmental perspective in psychiatry: what do recent genetic-epidemiological findings show? Mol. Psychiatry 25, 1631–1639. 10.1038/s41380-020-0648-1. 31959848 PMC7387296

[R69] Uliana DL, Gomes FV, Grace AA, 2021. Stress impacts corticoamygdalar connectivity in an age-dependent manner. Neuropsychopharmacology 46, 731–740. 10.1038/s41386-020-00886-3. 33096542 PMC8027626

[R70] Uliana DL, Gomes FV, Grace AA, 2022. Nucleus reuniens inactivation reverses stress-induced hypodopaminergic state and altered hippocampal-accumbens synaptic plasticity. Neuropsychopharmacology. 10.1038/s41386-022-01333-1. PMC9205859 35488085

[R71] Varela C, Kumar S, Yang JY, Wilson MA, 2014. Anatomical substrates for direct interactions between hippocampus, medial prefrontal cortex, and the thalamic nucleus reuniens. Brain Struct. Funct. 219, 911–929. 10.1007/s00429-013-0543-5. 23571778 PMC4179252

[R72] Vertes RP, 2002. Analysis of projections from the medial prefrontal cortex to the thalamus in the rat, with emphasis on nucleus reuniens. J. Comp. Neurol. 442, 163–187. 10.1002/cne.10083. 11754169

[R73] Vertes RP, Hoover WB, Szigeti-Buck K, Leranth C, 2007. Nucleus reuniens of the midline thalamus: link between the medial prefrontal cortex and the hippocampus. Brain Res. Bull. 71, 601–609. 10.1016/j.brainresbull.2006.12.002. 17292803 PMC4997812

[R74] Viena TD, Linley SB, Vertes RP, 2018. Inactivation of nucleus reuniens impairs spatial working memory and behavioral flexibility in the rat. Hippocampus 28, 297–311. 10.1002/hipo.22831. 29357198 PMC5871605

[R75] Wegrzyn D, Juckel G, Faissner A, 2022. Structural and functional deviations of the hippocampus in schizophrenia and schizophrenia animal models. Int. J. Mol. Sci. 23, 5482. 10.3390/ijms23105482. 35628292 PMC9143100

[R76] Wei H, Bonjean M, Petry HM, Sejnowski TJ, Bickford ME, 2011. Thalamic burst firing propensity: a comparison of the dorsal lateral geniculate and pulvinar nuclei in the tree shrew. J. Neurosci. 31, 17287–17299. 10.1523/JNEUROSCI.6431-10.2011. 22114295 PMC3236686

[R77] Wellman CL, Bangasser DA, Bollinger JL, Coutellier L, Logrip ML, Moench KM, Urban KR, 2018. Ses of repeated stress on age-dependent GABAergic regulation of the lateral nucleus of the amygdalion. J. Neurosci. 38, 9423–9432. 10.1523/JNEUROSCI.1673-18.2018. 30381434 PMC6209838

[R78] Wu YC, Du X, van den Buuse M, Hill RA, 2014. Sex differences in the adolescent developmental trajectory of parvalbumin interneurons in the hippocampus: a role for estradiol. Psychoneuroendocrinology 45, 167–178. 10.1016/j.psyneuen.2014.03.016. 24845187

[R79] Zhang W, Rosenkranz JA, 2016. Effects of repeated stress on age-dependent GABAergic regulation of the lateral nucleus of the amygdala. Neuropsychopharmacol 41, 2309–2323. 10.1038/npp.2016.33. PMC4946062 26924679

[R80] Zhang Y, Yoshida T, Katz DB, Lisman JE, 2012. NMDAR antagonist action in thalamus imposes δ oscillations on the hippocampus. J. Neurophysiol. 107, 3181–3189. 10.1152/jn.00072.2012. 22423006 PMC3378362

[R81] Zhu X, Grace AA, 2022. Sex- and exposure age-dependent effects of adolescent stress on ventral tegmental area dopamine system and its afferent regulators. Mol. Psychiatry. 10.1038/s41380-022-01820-3. PMC9918682 36224257

[R82] Zimmerman EC, Grace AA, 2016. The nucleus Reuniens of the midline thalamus gates prefrontal-hippocampal modulation of ventral tegmental area dopamine neuron activity. J. Neurosci. 36, 8977–8984. 10.1523/JNEUROSCI.1402-16.2016. 27559178 PMC4995308

[R83] Zimmerman EC, Grace AA, 2018. Prefrontal cortex modulates firing pattern in the nucleus reuniens of the midline thalamus via distinct corticothalamic pathways. Eur. J. Neurosci. 48, 3255–3272. 10.1111/ejn.14111. 30107061 PMC6237082

