# Supplementary material for: Age- and sex-dependent effects of stressors on activity of the nucleus reuniens of the thalamus
Source: Schizophr Res. Author manuscript; Available in PMC 2026 Jul 21. (PMC13387477; doi:10.1016/j.schres.2025.09.018)
Supplement: 1 [file NIHMS2183845-supplement-1.docx]

**Supplementary material**

Age and Sex-dependet effect of stressors on nucleus reuniens of the thamalus activity.

**Supplementary Table 1.** Mean ± SEM values for the number of neurons per track, firing rate, and % of spikes in bursts in the RE of experimental groups.

|  | **# of neurons**  **per track** | **Firing rate**  **(Hz)** | **% of spikes in burst** |
| --- | --- | --- | --- |
| **Early adolescence stress** |  |  |  |
| **1-2 weeks (PD47-54)** |  |  |  |
| Male - Naïve (n=6) | 2.22 ± 0.15 | 3.38 ± 0.53 | 34.41 ± 3.71 |
| Male - Early Stress (n=8) | 1.46 ± 0.29 | 2.53 ± 0.26 | 52.70 ± 4.11 |
| Female - Naïve (n=8) | 1.90 ± 0.23 | 3.63 ± 0.54 | 32.57 ± 3.59 |
| Female - Early Stress (n=8) | 1.60 ± 0.29 | 2.16 ± 0.24 | 47.47 ± 3.69 |
|  |  |  |  |
| **5-6 weeks (PD75-82)** |  |  |  |
| Male - Naïve (n=8) | 1.58 ± 0.18 | 2.22 ± 0.32 | 42.25 ± 3.76 |
| Male - Early Stress (n=8) | 1.31 ± 0.35 | 1.7 ± 0.26 | 35.5 ± 4.5 |
| Female - Naïve (n=8) | 0.69 ± 0.10 | 1.48 ± 0.27 | 21.44 ± 5.04 |
| Female - Early Stress (n=8) | 0.69 ± 0.09 | 2.46 ± 0.3 | 48. 09 ± 6.65 |
|  |  |  |  |
| **Late adolescence stress** |  |  |  |
| **1-2 weeks (PD57-64)** |  |  |  |
| Male - Naïve (n=7) | 1.53 ± 0.18 | 2.2 ± 0.23 | 35.23 ± 3.84 |
| Male - Late Stress (n=6) | 1.40 ±  0.25 | 2.54 ± 0.36 | 24.84 ± 3.87 |
| Female - Naïve (n=7) | 0.64 ± 0.07 | 2.24 ± 0.37 | 32.14 ± 5.77 |
| Female - Late Stress (n=7) | 1.27 ±  0.12 | 2.52 ± 0.38 | 34.09 ± 4.35 |
|  |  |  |  |
| **5-6 weeks (PD85-92)** |  |  |  |
| Male - Naïve (n=11) | 1.47 ± 0.18 | 2.67 ± 0.26 | 34.14 ± 2.97 |
| Male - Late Stress (n=13) | 1.12 ±  0.17 | 2.31 ± 0.25 | 32.76 ± 3.21 |
| Female - Naïve (n=9) | 0.46 ± 0.07 | 2.19 ± 0.29 | 42.98 ± 5.38 |
| Female - Late Stress (n=10) | 1.40 ±  0.21 | 2.61 ± 0.33 | 37.74 ± 3.69 |

**Supplementary Table 2**. RE proportion of neuron burst- and tonic-firing values (%) in the experimental groups.

|  | **Burst-firing neurons (%)** | **Tonic-firing neurons (%)** |
| --- | --- | --- |
| **Early adolescence stress** |  |  |
| **1-2 weeks (PD47-54)** |  |  |
| Male - Naïve (n=6) | 78,75 | 21,25 |
| Male - Early Stress (n=8) | 81,43 | 18,57 |
| Female - Naïve (n=8) | 71,43 | 28,57 |
| Female - Early Stress (n=8) | 85,71 | 14,29 |
|  |  |  |
| **5-6 weeks (PD75-82)** |  |  |
| Male - Naïve (n=8) | 79,49 | 20,51 |
| Male - Early Stress (n=8) | 66,67 | 33,33 |
| Female - Naïve (n=8) | 45,71 | 54,29 |
| Female - Early Stress (n=8) | 76,67 | 23,33 |
|  |  |  |
| **Late adolescence stress** |  |  |
| **1-2 weeks (PD57-64)** |  |  |
| Male - Naïve (n=7) | 86,67 | 13,33 |
| Male - Late Stress (n=6) | 69,77 | 30,23 |
| Female - Naïve (n=7) | 75 | 25 |
| Female - Late Stress (n=7) | 75 | 25 |
|  |  |  |
| **5-6 weeks (PD85-92)** |  |  |
| Male - Naïve (n=11) | 86,6 | 13,4 |
| Male - Late Stress (n=13) | 80,46 | 19,54 |
| Female - Naïve (n=9) | 81,25 | 18,75 |
| Female - Late Stress (n=10) | 72,41 | 27,59 |


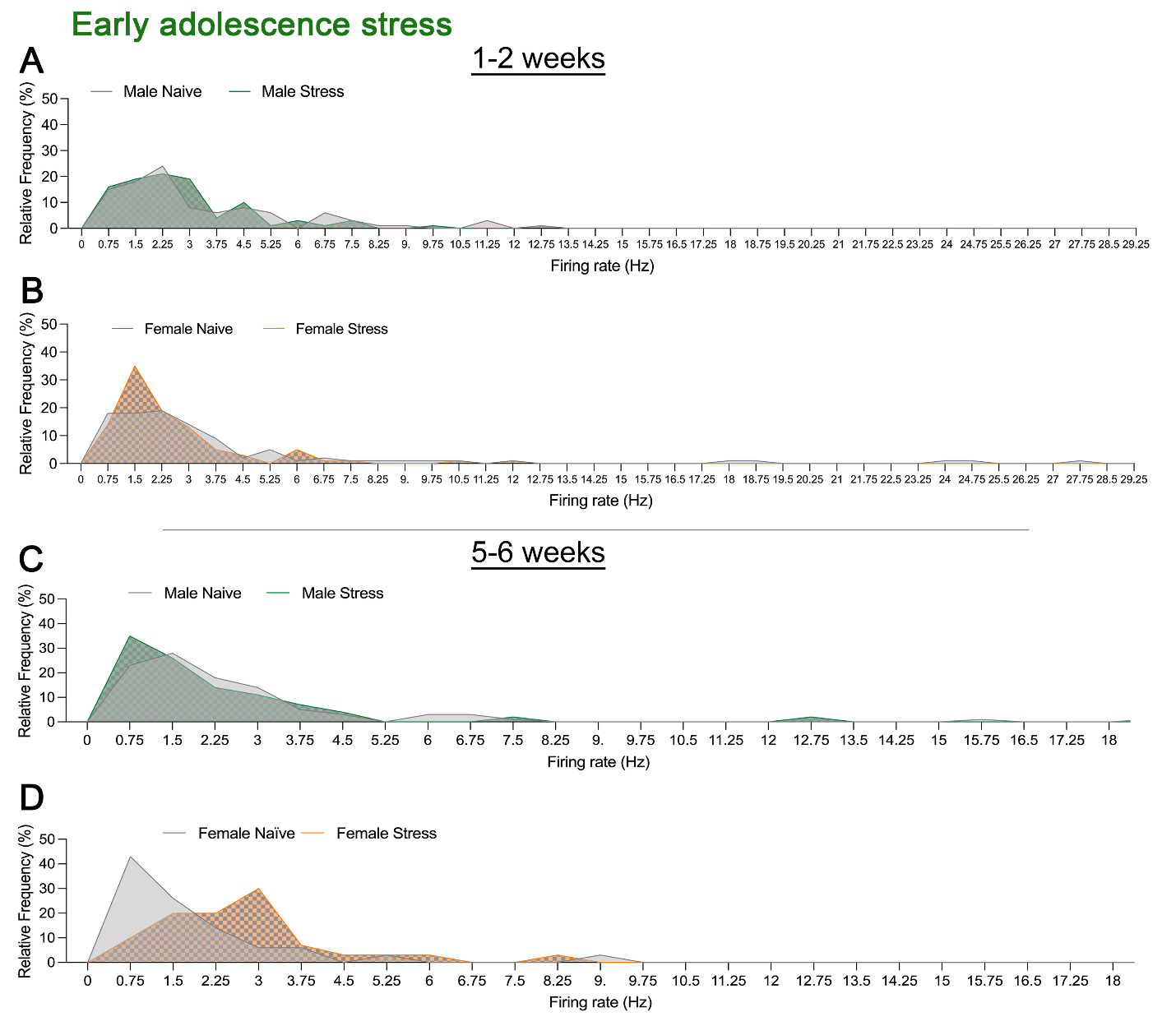


**Supplementary figure 1. Effect of early adolescence stress on firing fate distribution in males and females.** Early adolescence stress did not affect the firing rate distribution in either males or females at 1-2 weeks (A, B) or 5-6 weeks (C, D) post-stress.

**
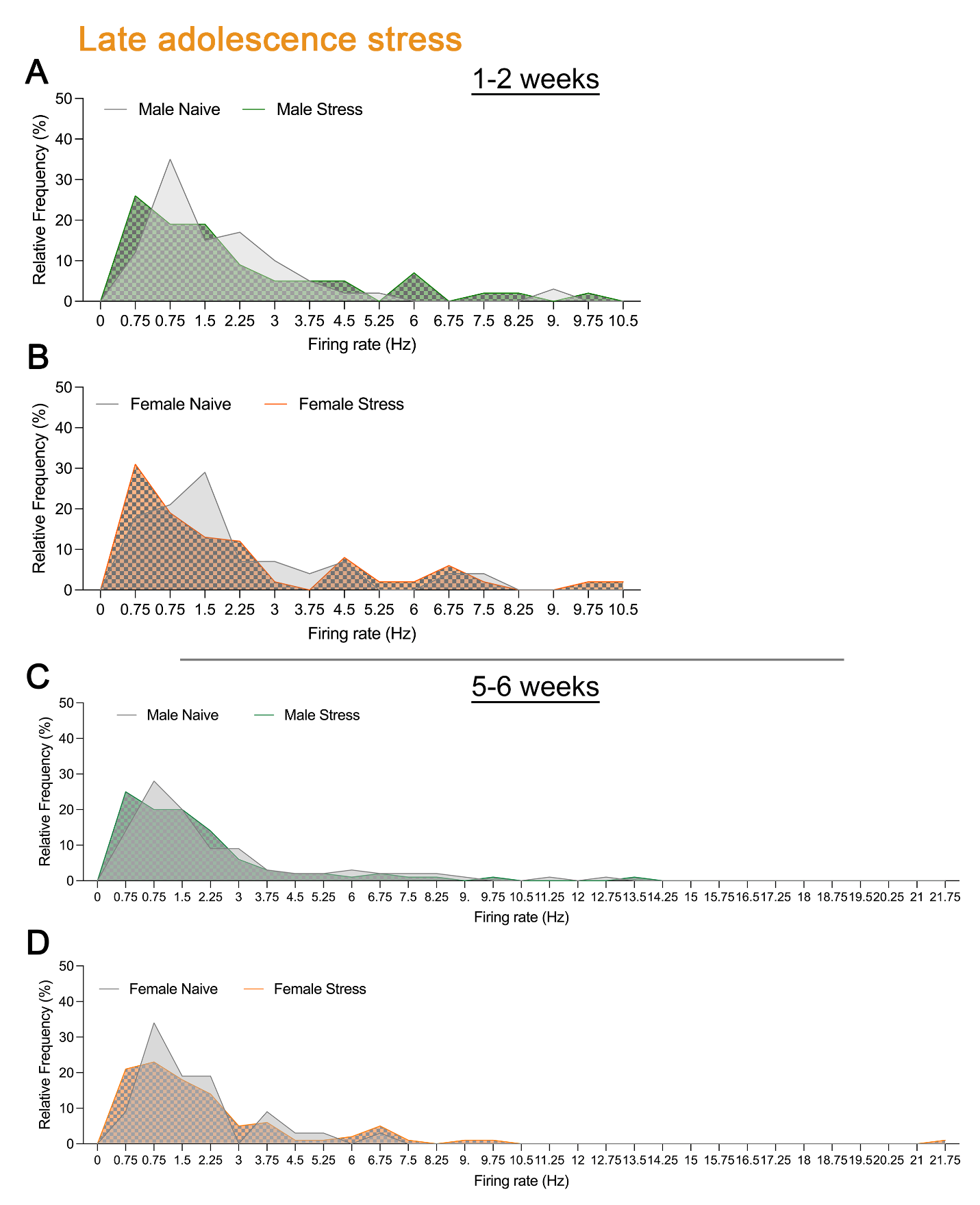
 Supplementary figure 2. Effect of late adolescence stress on firing rate distribution in males and females.** Late adolescence stress did not affect the firing rate distribution in either males or females at 1-2 weeks (A, B) or 5-6 weeks (C, D) post-stress.

**
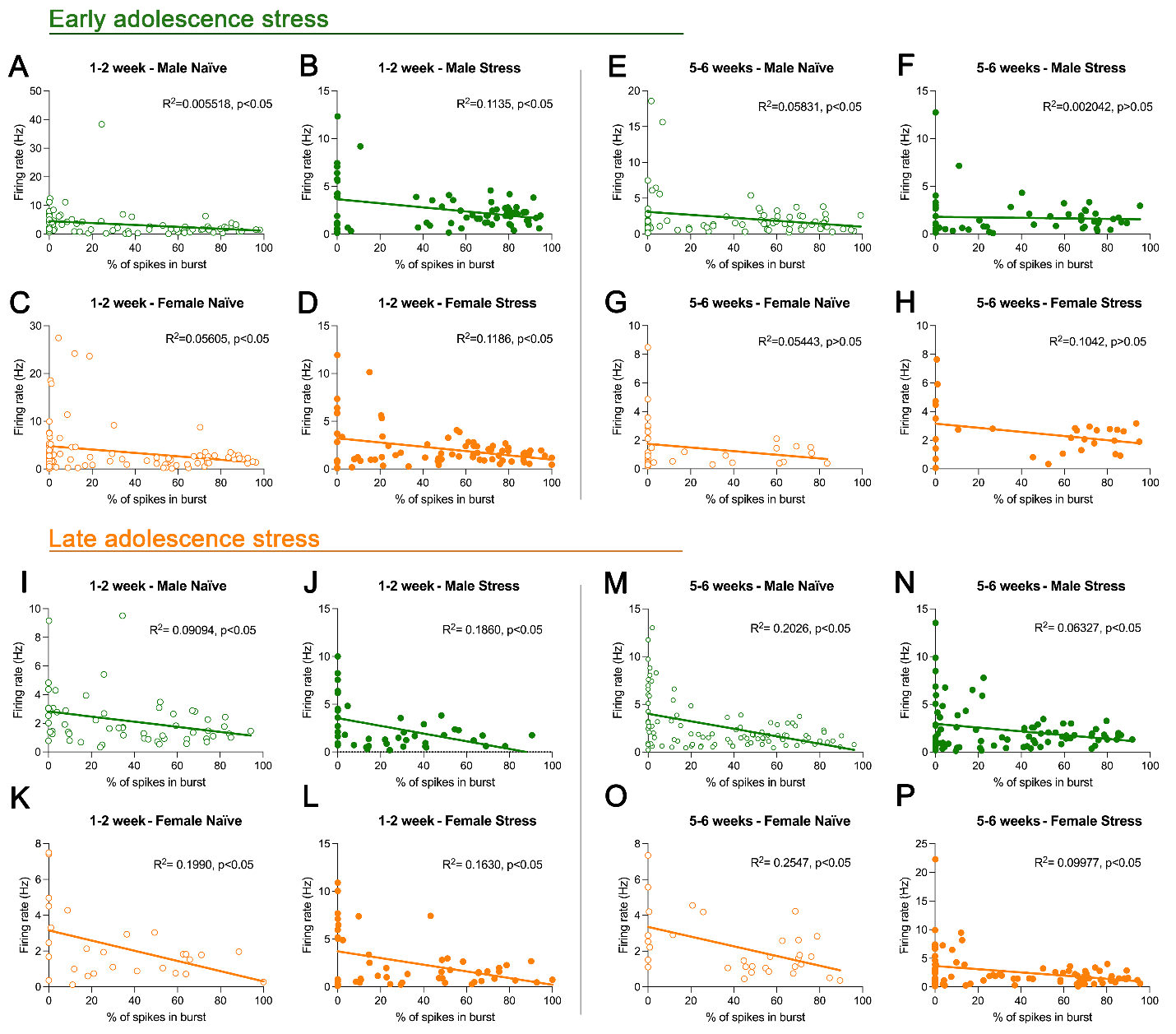
**

**Supplementary Figure 3. Correlation between firing rate and % of spikes in bursts in male and female rats subjected to early and late adolescence stress.** Correlation analysis revealed that RE neurons with lower firing rates are generally associated with increased burst-firing activity. This relationship was observed in the marjority of the groups, regardless of exposure to early adolescence (A-E) or late adolescence stress (I-P). However, exceptions were noted in stressed males, stressed females, and naïve females at 5-6 weeks post-early adolescence stress.

**
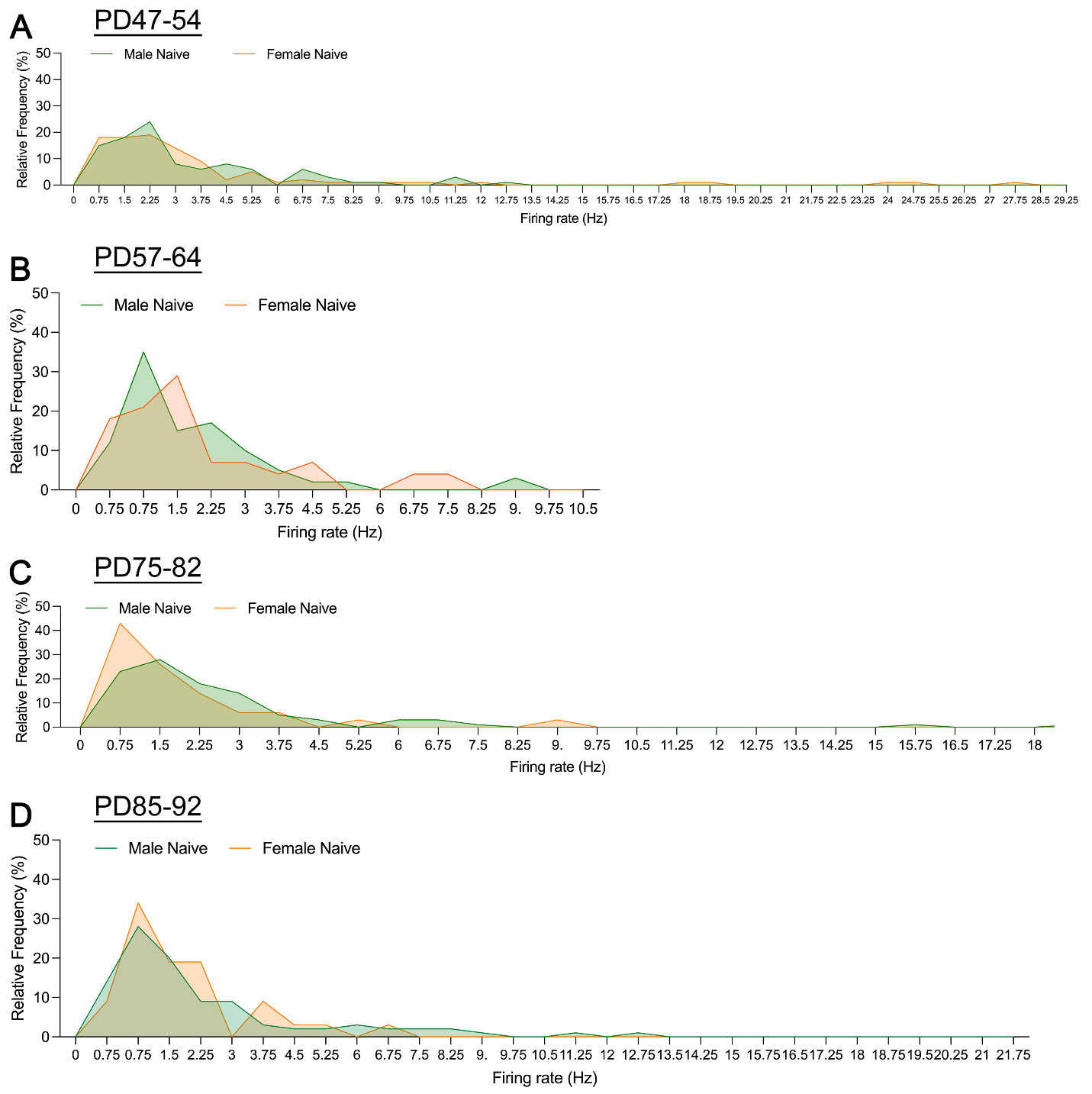
**

**Supplementary Figure 4. Effect of age on firing rate distribution in males and females.** The firing rate distribution of RE neurons did not differ between males and females during PD47-54 (A), PD57-64 (B), and PD85-92 (D). However, during PD75-82, females exhibited a greater proportion of RE neurons firing at low frequencies (0-1.5 Hz) compared to males (C; Kolmogorov-Smirnov, D = 0.49, p < 0.05).

**
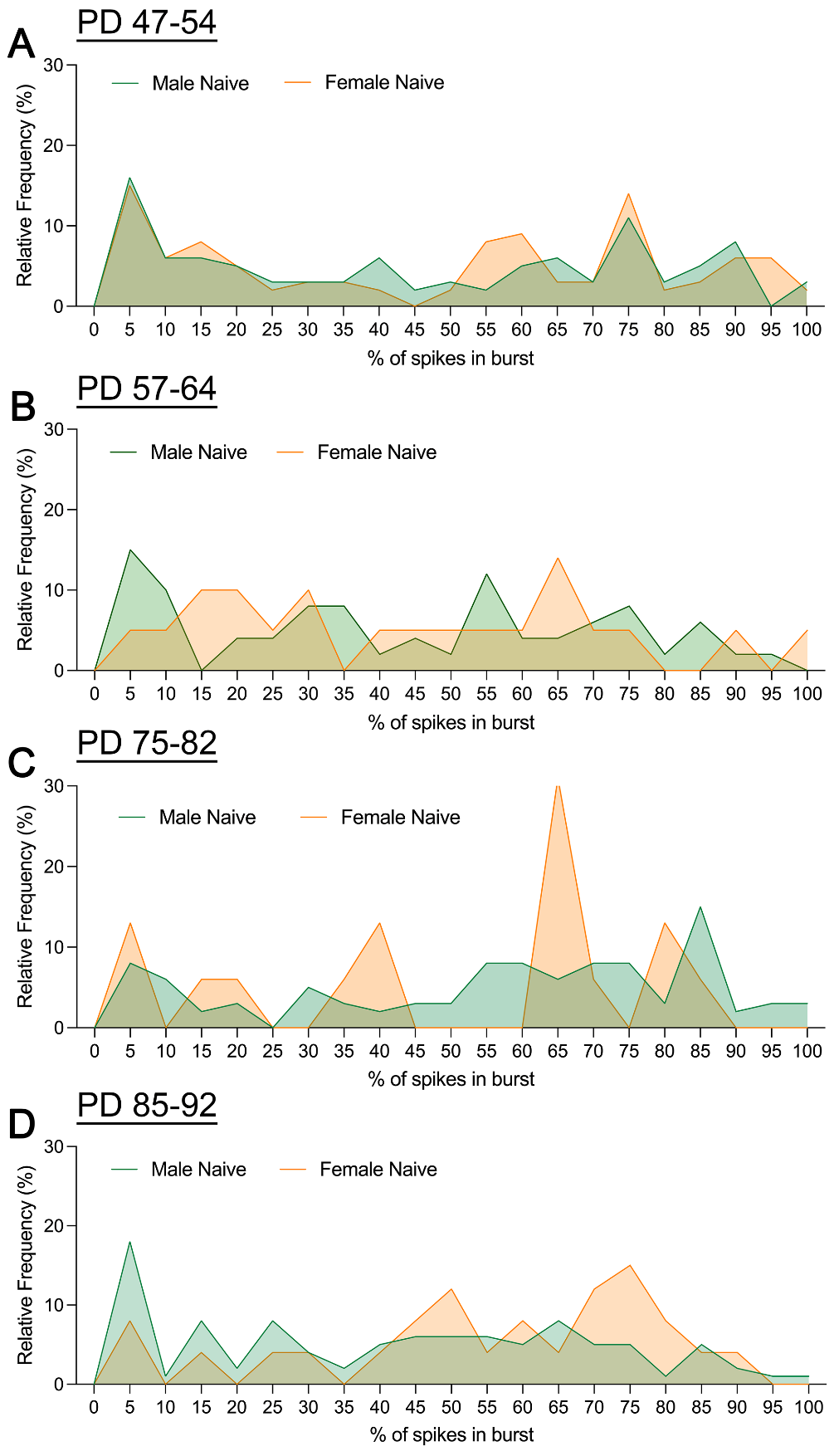
**

**Supplementary Figure 4. Effect of age on the % of spikes in bursts in males and females.** The % of spikes in bursts among RE neurons exhibiting burst-firing properties did not differ between males and females across the examined age periods of PD47-54 (A), PD57-64 (B), PD75-82 (C), and PD85-92 (D).
